# Unmet need for family planning and associated factors among currently married women of reproductive age in Bishoftu town, Eastern Ethiopia

**DOI:** 10.1371/journal.pone.0260972

**Published:** 2021-12-06

**Authors:** Megersa Girma Garo, Sileshi Garoma Abe, Worku Dugasa Girsha, Dawit Wolde Daka

**Affiliations:** 1 Bishoftu Defence Hospital, Bishoftu, Ethiopia; 2 Adama Hospital Medical Science College, Adama, Ethiopia; 3 Faculty of Public Health, Jimma University, Jimma, Ethiopia; University of Western Australia, AUSTRALIA

## Abstract

**Background:**

Unmet family planning is one of the common causes for low contraceptive prevalence rates in developing countries, including Ethiopia. Rapid urbanization had profound effect on population health, however, little is known about the unmet need of family planning in settings where there was increased industrializations and internal migrations in Ethiopia. This study aims to determine the unmet need for family planning services among currently married women and identify factors associated with it in Bishoftu town, Eastern Ethiopia.

**Methods:**

Community-based cross-sectional study was conducted from 1^st^ January to 28^th^ February, 2021 among 847 randomly sampled currently married women of the reproductive age group. Data were collected using semi structured interviewer administered questionnaire. Multivariate logistic regression was used to identify factors associated with the outcome variable and a 95% confidence interval was used to declare the presence of statistical significance associations.

**Results:**

Eight hundred twenty-eight women were participated in the study. The prevalence of unmet need for family planning among currently married women was 26% [95% CI: 23,29]. Maternal age [AOR, 3.00, 95% CI:1.51–5.95], educational status [AOR, 2.49, 95% CI:1.22–5.07], occupational status of self-employee [AOR, 1.98, 95% CI:1.15–3.39] and housewife [AOR, 1.78, 95% CI:1.02–3.12], being visited by health care provider in the last 12 months [AOR, 1.81, 95% CI: 1.26–2.60] and desired number of children less than two [AOR, 1.53, 95% CI:1.01–2.30] were significantly associated with unmet need for family planning.

**Conclusions:**

Unmet need for family planning was higher in the study area compared with the United Nations sphere standard of unmet need for family planning and the national average, and slightly lower than the regional average. Socio-demographic, economic, and health institution factors were determinants of the unmet need for family planning in the study area. Therefore, health education and behaviour change communication related to family planning services should be strengthened and access to family planning services should be improved.

## Introduction

Unmet need in family planning is defined as the percentage of women who don’t want to be pregnant but are not using contraception. It indicates the gap between childbearing desire and contraceptive use, and calculated as the percentage of women of reproductive age, either married or in union, who have an unmet need for family planning [1].

Globally the prevalence of contraceptive use is increasing, however the unmet need for contraception remains a problem [2,3]. In 2019, 190 million (10%) of married women are estimated to have an unmet need for family planning. The prevalence of unmet need was higher in Africa (22%) and Oceania region (15%) compared to other parts of the world. Unmet need for contraceptives among married women was estimated at or below 10% [3,4].

Family planning services have a substantial impact on maternal morbidity and mortality. A rise in contraceptive prevalence rate and the decline in unmet need for family planning significantly reduces maternal mortality and disability associated with complications of pregnancy and child birth [5–11]. Despite this fact, unacceptably millions of women suffer from unintended pregnancies (both mistimed and unwanted) and related consequences worldwide every year [9–12].

Ethiopia is one of the twenty-two countries with a high maternal mortality rate worldwide in 2017 with maternal mortality rate ranging between 300 and 999 per 1000 live births. The maternal mortality rate in 2017 was 401 per 1000 live births [13]. The prevalence of unintended pregnancy in Ethiopia was 28% and Oromia region (33.8%) accounted the highest prevalence followed by Southern Nations, Nationalities, and People’s region (30.6%). Satisfying demands for family planning leads to fewer unintended pregnancies, abortions, and child and maternal deaths [14].

The current level of unmet need for family planning was unacceptability high in Ethiopia standing at 22% in 2020 that is far away from the target set by the country (10%) [15]. Unmet needs for family planning varies with socio-demographic factors, residence, and geographic regions, with the highest unmet needs being in the Oromia region accounting to 29%. Unmet need for family planning had declined steadily in the period between 2000 to 2016 [16] and the prevalence of contraceptives had increased in the period from 2000 (8%) to 2019 (44%) [17].

Proper understanding of the extent of an unmet need for family planning and the factors associated with it has paramount importance in addressing the problems related to non-use of family planning services. It has substantial contributions to improve the health conditions of women and children and the underlying socio-economic problems of the country. There were studies that investigated the unmet need for family planning and associated factors in Ethiopia [18–25], and little evidence exists regarding unmet need for family planning particularly in settings where there was growing industrialization and increased internal migration. Rapid urbanization had profound effect on population health [26]. There may be contextual differences among the study participants in different settings. Therefore, this study aimed to determine the unmet need for family planning services and identify factors associated with it among currently married women in Bishoftu town, Eastern Ethiopia. The study was focused in urban setting with a growing industrialization and aimed to identify socio-demographic and economic factors, reproductive health factors, and service characteristics that predict an unmet need for family planning among currently married women.

## Methods and materials

### Study setting and period

The study was conducted in Bishoftu town. Bishoftu town is one among the town administrations of Oromia region located 47 KM far away from Addis Ababa in Eastern part of Ethiopia. It is located adjacent to Eastern industrial zone of Ethiopia. The town administration comprises of 14 kebeles of which nine were urban kebeles and the remaining three were semi-urban kebeles. In the town, there were two public hospitals, five health centers, two private hospitals, and ten private clinics providing services ranging from preventive and basic curative to advanced medical services to the catchment area population. According to Bishoftu town health office report of 2020, the town had a total population of 217,971 and of this female population accounted 51% (111,165) and women in the reproductive age group eligible to family planning services were 19% (41,328). The study was conducted from 1^st^ January to 28^th^ February, 2021.

### Study design and participants’ selection

A community-based cross-sectional study was conducted among currently married women of reproductive age group. All currently married women in the reproductive age group (15–49) and lived in the town for at least 6 months were eligible to the study. Infecund women, women who were not legally married and who were critically ill during the survey period were excluded. Infecundity and marital status of women were identified based on self-report.

The sample size was determined using Epi Info sample size calculator for cross-sectional surveys considering the assumptions and parameters: 95% confidence level, 4% margin of error, proportion of unmet need for family planning as 30.9% from the study conducted in Debre Birhan [27], 1.5 design effect, and 10% non-response rate. The calculated sample size yields 846.


$$Sample\;size\;(n)=\frac{\left(Z^{2}*P*Q\right)}{d^{2}}$$

Where:

■ Confidence level (Z) = 1.96
■ Prevalence of outcome in the population (P) = 30.9%
■ Q = 1-P = 69.1%
■ Margin of error (d^2^) = 4%

**Fig 1:**
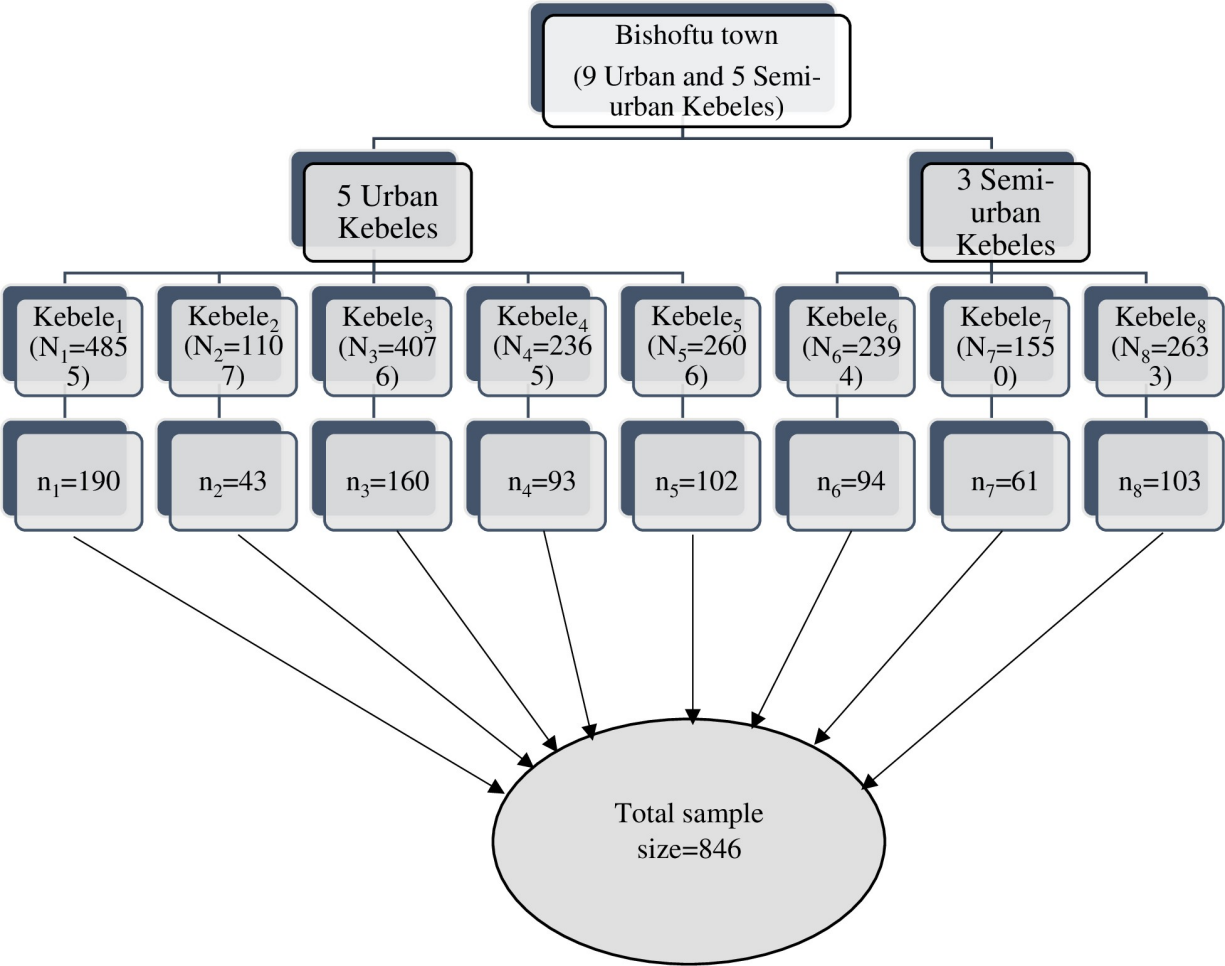
Sample size and sampling strategy.

Multi-stage stratified sampling strategy was used to select kebeles and study participants. In the first stage, five urban kebeles and three semi-urban kebeles were selected using simple random sampling strategy from nine urban and five semi-urban kebeles. In the second stage, a systematic random sampling strategy was used to select households in each kebeles. All households in the primarily selected Kebeles and women in the reproductive age group were listed. The sample size was proportionally allocated to each selected Kebeles based on the total number of currently married women in each Kebeles. All eligible women in every 25^th^ household (K = 25) were interviewed after taking consent for participation. In the case when the selected households had no eligible women, the next household was considered and whenever there were more than one eligible women in the sampled household, one woman was selected randomly. Each selected household was visited three times on occasions where respondents were unavailable during the first visit and after the third visit households were recorded as no-response (Fig 1).

### Study variables

The outcome variable was the unmet need for family planning. It was the sum of the unmet need for spacing and the unmet need for limiting. Other outcome variables included demand for family planning and demand satisfied for family planning. Demand for family planning was calculated as the sum of currently married women who were on family planning and the unmet need for family planning. Percentage of demand satisfied for family planning was calculated as currently using family planning divided by the demand for family planning. The explanatory variables were socio-demographic and economic factors, reproductive health factors, and service characteristics.

### Data collection tools and procedures

Semi structured interviewer administered questionnaire was used to collect data. The questionnaire was developed based on relevant literature and adapted to the research context [21,23,27,28]. It comprised of three parts: background of respondents, reproductive characteristics, and service related factors. The questionnaire was primarily developed in English language and translated to local languages (Afan Oromo and Amharic).

Five nurses and two supervisors with qualification of bachelor of science in public health were participated in the data collection process. A two-day training was given to data collectors and supervisors on the questionnaire, data collection process and research ethics. Pre-test was conducted in 5% of the sample size in an adjacent Kebele of the study area and corrections were made to the questionnaire as appropriate. Supervisors have closely monitored the data collection process and provided support at the field level.

### Data processing and analysis

Each data records were checked for completeness and consistency, and duplicated cases were removed. Data were entered to Epi-Info version 7 and exported to SPSS version 25 for analysis. The data analysis was progressed in such a way that primarily descriptive statistics was used to describe and summarize the characteristics of respondents. Secondly, bivariate logistic regression was undertaken and those variables with P-value<0.25 were taken to multivariate logistic regression. The outcome variable of the study was the unmet need for family planning services. Variable Inflation Factor (VIF) was used to check the presence of multi collinearity and Hosmer-Lemeshow test of Goodness-of-fit was used to test how well the model explains the data. The strength of association was expressed in an odds ratio with 95% confidence interval and P-value <0.05 was used as cut-off point to declare significance in the final model.

### Ethical consideration

Research ethical clearance was obtained from Adama Hospital Medical College Institutional Research Ethics Review Board (Reference number: AHMC/MPHWek/8/12/2020). Support letter was taken from Oromia Regional Health Bureau and Bishoftu town health office. The research was conducted according to the Declaration of Helsinki. The research aims, benefits, and risks were explained to each research participant. Following this, a written informed consent was obtained from participants and for minors, informed written consent was taken from parents or guardians. No personal identifiers were recorded and codes were used on each questionnaire. Paper based data was kept in a locked cabinet and computer-based data were secured with a confidential password. Research data will only be used for the intended aim and not shared with the third people.

## Results

Eight hundred twenty-eight currently married women in the reproductive age group were participated in the study with a response rate of 97.8%.

### Sociodemographic and economic characteristics of women

The mean age of women was 31 years (SD 6.6) with a minimum age of 17 years and maximum age of 49 years. Four in ten (42%) of women were in the age group of 26–35 followed by women aged less than 25 years. Higher proportion of women were Orthodox Christians (36%) and had higher educational status (38%) (Table 1).

**Table 1:** Socio-demographic and economic characteristics of women in Bishoftu town, Eastern Ethiopia, January 2021.

Variable	Frequency (N = 828)	Percent
Age in years		
**≤**25	224	27
26–35	350	42
36–45	174	21
≥46	80	10
Ethnicity		
Oromo	395	48
Amhara	217	26
Tigre	85	10
Somali	69	8
Guraghe	56	7
Others*	6	1
Religion		
Orthodox	297	36
Muslim	129	16
Protestant	176	21
Wakefata	122	15
Catholic	104	13
Educational status		
No education	166	20
Primary	91	11
Secondary	257	31
Tertiary	314	38
Occupational Status		
Government employee	109	13
Self-employee	280	34
Merchant	112	14
House wife	242	29
Daily labourer	85	10
Monthly income in Ethiopian Birr(ETB)		
**≤**2500	254	31
2500–5000	299	36
5001+	275	33

*Others: Kabata (n = 3), Wolayita (n = 3).

### Reproductive health characteristics

Age at first marriage was as early as 17 years and as late as 35 years with a mean age of 22 years (SD 3.3). Mean age at first pregnancy was 18 years. Thirty-eight percent of women had a desire to have two and fewer children and higher than half (54%) of them had 1–2 living children. Twelve percent of women were currently pregnant with 8% of them wanting the pregnancy now, 2% wanted the pregnancy latter, and 2% not wanted the pregnancy at all (Table 2).

**Table 2:** Reproductive health characteristics of currently married women of reproductive age in Bishoftu Town, Eastern Ethiopia, January 2021.

Variable	Frequency (N = 828)	Percent (95% CI)
Age at first marriage in years		
**≤**19	262	32(28.5–34.9)
20–24	316	38(34.9–41.5)
≥25	250	30(27.1–33.4)
Age at first pregnancy in years (N = 719)		
**≤ **19	52	7(5.5–9.3)
20–24	449	62(58.9–65.9)
≥25	218	30(27.0–33.8)
Desired number of children		
**≤ **2	313	38(34.5–41.2)
3–4	259	31(28.2–34.5)
5+	256	31(27.8–34.1)
Number of living children		
No child	133	16(13.7–18.7)
1–2	444	54(50.2–57.0)
3–4	236	29(25.5–31.7)
5+	15	2(1.1–2.9)
Currently pregnant		
Yes	96	12(9.6–13.9)
No	732	88(86.1–90.5)
The current pregnancy was (N = 96)		
Wanted now	63	66(55.7–74.6)
Wanted latter	19	20(12.7–28.7)
Not wanted at all	14	15(8.6–22.7)

Abbreviations: *CI* confidence interval.

### Current contraceptive use patterns and service characteristics

Four hundred thirty-five (53%) women were using contraceptives at the time of the survey and out of which 107 (25%) and 328(75%) of the women were using contraceptives for limiting and spacing purposes, respectively. The most commonly used family planning method was implants 214(49%) followed by injectable 110(25%) and IUCD 84(19%). Pills accounted of 20(5%) and condoms accounted of 7(2%).

Slightly greater than half (54%) of women were visited by health care providers in the last 12 months of the survey period, and the round trip to visit a health institutions was less than or equals to 30 minutes for most (98%) of the women surveyed. Four-hundred sixty (56%) of women spent less than 15 minutes in health institutions and greater than half (53%) of women told about family planning options in their current visits. Appointment was given to 52% of women (Table 3).

**Table 3:** Family planning service characteristics among currently married women of reproductive age in Bishoftu Town, Eastern Ethiopia, January 2021.

Variable	Frequency	Percent (95% CI)
Visited by Family planning provider in the last 12 months		
Yes	444	54(50.2–57.0)
No	384	46(43.0–49.8)
Time taken for round trip for Family planning service		
≤ 15 minutes	402	49(45.2–52.0)
16–30 minutes	410	49(46.1–52.9)
31–60 minutes	16	2(1.2–3.1)
Time taken in the health facility		
≤15 minutes	460	56(52.2–58.9)
16–30 minutes	256	31(27.8–34.1)
31–60 minutes	112	14(11.3–16.0)
Discussed about family planning options		
Yes	442	53(50.0–56.8)
No	386	47(43.2–50.0)
Got family planning method wanted		
Yes	516	62(59.0–65.6)
No	312	38(34.4–41.0)
Provider give appointment in the current visit		
Yes	433	52(49.0–55.7)
No	395	48(44.3–51.1)

Abbreviations: *CI* confidence interval.

### Unmet need for family planning

Unmet need for family planning was calculated by summing up the number of women who do not use family planning because of fear of side effects, not having sex, infrequent sex, and health concerns, but they want the child later and the other did not want at all.

**Fig 2:**
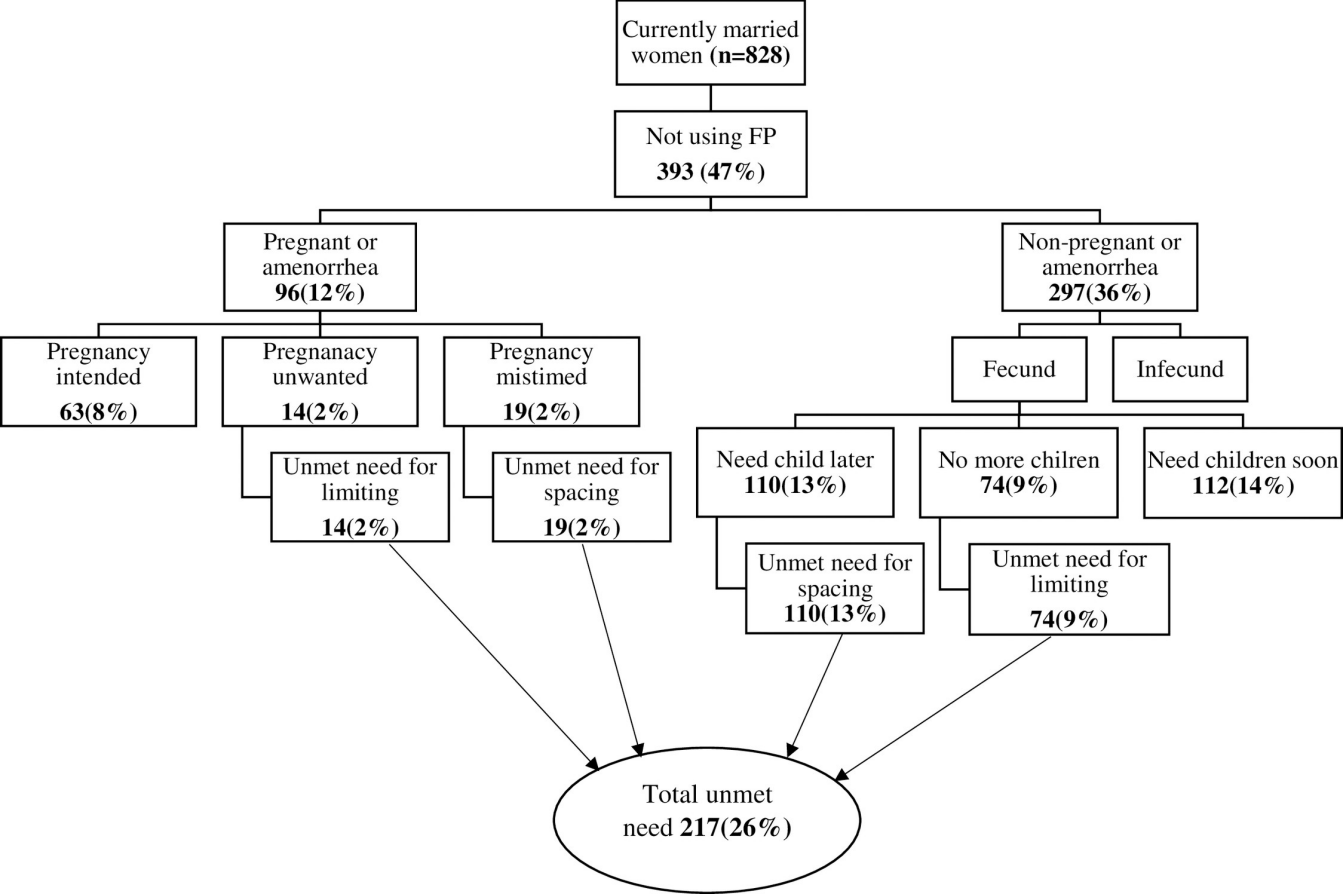
Schematic diagram of the unmet need for family planning among currently married women.

Unmet need for family planning among currently married women in reproductive age groups was 217 [26%, 95% CI: 23–29]. Out of this, 129 (16%) were unmet need for spacing and 88(11%) were unmet need for limiting. Demand for family planning in the study area was the sum of currently on family planning 435(53%) and current unmet need for family planning 217(26%), which was 79%. Percentage of demand satisfied for family planning was 67% (that is currently on family planning (n = 435) divided by demand for family planning (n = 652) (Fig 2).

The major reason of not using the family planning method was wanting more children 150(38%) followed by not having sex due to their husbands were on field work 102(26%). Fear of side effects accounted of 59(15%), infrequent sex accounted of 50(13%), health concern accounted of 24(6%), and menopause/ hysterectomy accounted of 12(3%).

### Factors associated to the unmet need for family planning

In the bivariate logistic regression analysis, maternal age, educational status, occupation status, total birth, desired number of children, income, age at first pregnancy, number of pregnancies, being visited by a health care provider in the last 12 months, main place where they use family planning, and age at first marriage were identified as candidate variables with P-value<0.25.

In the multivariate logistic regression analysis, only variables including maternal age, educational status, occupational status, desired number of children, and being visited by a health care provider in the last 12 months were statistically associated with an unmet need for family planning after controlling other confounders.

The interpretations were given as below. The odds of unmet need for family planning was three times more likely among women with age less than 25 years compared to women with age greater than 46 years [AOR = 3.0, 95% CI:1.51–5.95]. The odds of unmet need for family planning was 2.5 times more likely among women with primary education compared to women with tertiary education [AOR, 2.49, 95% CI: 1.22–5.07]. The odds of unmet need for family planning was two times more likely among self-employed women [AOR, 1.98, 95% CI: 1.15–3.39] and house wife’s [AOR, 1.78, 95% CI: 1.02–3.12] compared to daily labourers, respectively. Women who had not been visited by health care providers within the last 12 months prior to the survey date were two times more likely to have an unmet need for family planning when compared to women who had been visited [AOR, 1.81, 95% CI: 1.26–2.60]. The odds of unmet need for family planning was 1.53 times more likely among women who wanted to give birth of ≤2 children compared to women who wanted five and more children [AOR, 1.53, 95% CI: 1.01–2.30] (Table 4).

**Table 4:** Factors associated with the unmet need for family planning.

Variables	Unmet need for FP		COR (95% CI)	AOR (95% CI)
	Yes (%)	No (%)		
Maternal age in years				
≤ 25	24(2.9)	200(24.2)	1.20[1.00–3.49]	3.00 [1.51–5.95] *
26–35	114(13.8)	236(28.5)	2.23[1.63–3.46]	0.83[0.47–1.47]
36–45	59(7.1)	115(13.9)	0.9[(0.99–1.23]	0.73[0.39–1.35]
≥ 46	20(2.4)	60(7.2)	1.0	1.0
Occupational status				
Government employee	33 (4)	76(9.2)	0.67[0.37–1.22]	1.65[0.87–3.13]
Self-employee	68 (8.2)	212(25.6)	1.49 [1.18–2.86]	1.98[1.15–3.39]*
Merchant	29 (3.5)	83(10)	0.54[0.29–0.99]	1.18[0.61–2.29]
House wife	54 (6.5)	189(22.8)	1.44 [1.26–2.75]	1.78[1.02–3.12]*
Daily labourer	33 (4)	51	1.0	1.0
Educational status				
No education	53 (6.4)	113 (13.6)	1.04 [0.58–1.86]	0.78[0.49–1.21]
Primary education	12 (1.4)	79 (9.5)	1.71 [1.0–2.91]	2.49[1.22–5.07]*
Secondary education	76 (9.2)	181 (21.9)	0.77 [0.53–1.13]	0.68[0.45–1.03]
Collage and above	76(9.2)	238(28.7)	1.0	1.0
Being visited by health care provider in the last 12 months				
No	102(12.3)	340(41.1)	3.71[2.66–5.17]	1.81[1.26–2.60]*
Yes	115(13.9)	271(32.7)	1.0	1.0
Number of children desired				
≤2	64(8.1)	249(30.2)	1.79[1.49–2.27]	1.53[1.01–2.31]*
3–4	81(9.4)	178(21.4)	0.21[0.13–0.35]	1.14[0.76–1.72]
5+	72(8.7)	184(22.2)	1.0	1.0

* Statistically significant at *P-value* < 0.05.

Abbreviations: *COR* crude odds ratio, *AOR* adjusted odds ratio, *CI* confidence interval, 1.0 reference category.

## Discussion

The study showed that a quarter of currently married women in the reproductive age group (26%, 95% CI: 23,29) had an unmet need for family planning in the study area. Sixteen percent of women had an unmet need for spacing and one in ten of women had an unmet need for limiting. Women age, occupational status, educational status, desired number of children, and being visited by a health care provider in the last 12 months were statistically associated with an unmet need for family planning.

The prevalence of unmet need for family planning in the study area was higher than the national average (22%) and slightly lower than the regional average (29%) [16]. It is far from the target set at national level that is 10% in the year 2020 [15]. The prevalence was higher compared with the United Nations sphere standard of unmet need for family planning, which is considered to be high if greater than 25% and the global estimate of unmet need for family planning among women in the reproductive age group (24.3%) [3,19]. The finding was also higher than studies conducted in different parts of the world including Zambia (21%) [29], Myanmar (19.4%) [30] and Bangladesh (13.5%), [31] and Kutaye district, Western Ethiopia (23.1%) [22]. Whereas, the finding was comparable to cross-sectional studies conducted in Tiro Afata (26%) [23], Misha (26.5%) [32], and Damot Woyde Woredas (26.3%) [20].The variations in the unmet need for family planning might be related to contextual variations including health services coverage, knowledge and attitudes towards family planning services, and socio-demographic and economic factors. On the contrary, studies conducted in Hargeisa, Somaliland (30%) [33], India (55.3%) [34], and Asebot town (37%) [21] and Debre Birhan town (31%) [27] in Ethiopia have shown a higher unmet need prevalence compared to the study area. Likewise, a systematic review and meta-analysis on studies done in different parts of Ethiopia showed slightly higher rate of unmet need for family planning among married women (32.8%) [25] and among women in the reproductive age group that ranged from 26.5% to 36.4% [19]. The higher prevalence of unmet need for family planning might be because of low access to contraceptives, low awareness towards modern contraceptive methods, and low partner involvement in such matters [18,35].

Demand for family planning was satisfied for two-thirds of currently married women. This was slightly higher than the national average (61.6%) and higher than Oromia region (48.9%) [16]. In the study area, only 53% of currently married women were using contraceptives and the majority of them (75%) use contraceptives for spacing purposes. The most commonly used methods of contraceptives were implants, injectable and IUCD, accounting of 93%. This was consistent to the national survey [36]. If all currently married women who said that they wanted to space and limit their children were to use family planning methods, the contraceptive prevalence in the study area would increase from 53% to 79%. The main reasons for non-use of family planning among currently married women were wanting more children, not having sex, fear of side effects, infrequent sex, health concern, and menopause, which is a result comparable with other studies else [24,37]. Thus, providing a comprehensive information of family planning methods enhances clients to use the most suitable methods and helps to attain SDG 3.7 of informed choice of contraceptive methods [38].

Unmet need for family planning has consequences on women and their families such as unintended pregnancy that in turn leads to increased maternal morbidity and mortality. It also leads to physical abuse and affects the overall wellbeing of women. Hence, addressing the family planning needs of women is critical in the effort towards attaining a goal related to maternal mortality [38,39]. In Ethiopia in the past two decades, the prevalence of contraceptives among currently married women has increased, although not as intended and stood at 41% in 2019 [17]. Unmet need for family planning had declined steadily from 36% in 2000 to 22% in 2016 [16]. This implies the need to accelerate efforts towards Sustainable Development Goals related to family planning.

Our study showed that factors such as socio-demographic, economic, reproductive health, and being visited by a health care providers were significantly associated with the unmet need for family planning. The odds of unmet need for family planning were more likely in the younger age group of currently married women than the older age group women. This finding was comparable to other parts of the world whereas in contrast to studies done in different parts of Ethiopia [21,28,29,31,40,41]. This might be because older women are mature and better decide on their health including the use of family planning services compared to younger women. Besides, older woman had less desire to children compared to younger women, which increases the need to use family planning methods. As the consequence of this a younger age groups of women should be targeted for family planning services in the study area.

The study also indicated the relationship between maternal education and occupational status with an unmet need for family planning. Odds of unmet need for family planning were more likely in women with primary education than college and above. This finding was comparable to a study conducted in different parts of the World and in Ethiopia [20,22,29,33,40,42]. Moreover, the odds of unmet need were more likely among self-employed women and housewife’s compared to daily labourers. This might be because self-employed women and those who were housewives are relatively stable and better decide on their preferences compared to daily labourers, which usually struggles to maintain their daily needs. Daily labourers are more likely to have control on their life situations with the number of children and accept and implement health actions related to family planning better than their counterparts. This finding was comparable to a study conducted in different parts of Ethiopia [25,27].

In the study area, women who were visited by a health care provider in the last 12 months of the survey date more likely had met the need for family planning services compared to women who were not visited. This is consistent with a study done in other settings of Ethiopia [22,23,27,41]. One of the service delivery modalities in the urban health extension program of Ethiopia is home to home or outreach services. Using this modality, the health extension professional provides health education and behaviour change communication related to family planning and other packages of services. They also provide family planning services to women who are less accessible to health facilities. The increased awareness and knowledge of women towards family planning services enable them to have access to services and hence, met needs [43–45].

Desire to have children was the other factor that affects the unmet need for family planning among currently married women. The odds of an unmet need for family planning was more likely among women who desired to have fewer children (**≤** 2 children) than those who desired to have more children (more than 5 children). As the number of children increases, the probability of using family planning services will increase. This finding is consistent with other studies [20–23,29,33,40].

The study has included a larger sample sizes and this enables appropriate generalisation of findings. The study was focused only on currently married women and doesn’t able to capture the important factors that determine the unmet need for family planning in other groups such as women who are in union and not married.

## Conclusions

Though modern contraceptive is a highly cost-effective public intervention in reducing maternal mortality [8,46,47], the current study indicated that the unmet need for family planning among currently married women was higher compared to the national average and slightly lower than the regional average. This has considerable implications on women and the society at large, including unintended pregnancy, unsafe abortions, physical abuse, and overall wellbeing. It increases maternal morbidity and mortality, and affects efforts toward improving the health status of women and the economic development of the nation.

Unmet need for family planning was associated with socio-demographic factors (maternal age), economic factors (educational and occupational status), desire to have children, and access to a health care provider. Hence, an effort should be made to strengthen access to family planning methods and health care providers in the study area. Health education and behaviour change communication strategies should be accessible to target women in the study area. This can be done by using health extension professionals and strengthening the urban health extension program, which has been under implementation since 2009 in Ethiopia. It also requires strengthening the women development army at the grass root level that helps to identify barriers to service utilization and enable access to health services through providing health information.

## Supporting information

S1 File (ZIP)

S1 Dataset (XLSX)

## References

[1] United Nations, Department of Economic and Social Affairs, Population Division. United Nations, Department of Economic and Social Affairs, Population Division (2014).World Contraceptive Use 2014 (POP/DB/CP/Rev2014). 2014. p. 1–3.

[2] United Nations Department of Economic and Social Affairs PD. World Fertility and Family Planning 2020: Highlights (ST/ESA/SER.A/440). World Fertility and Family Planning 2020: Highlights. 2020.

[3] Kantorová V, Wheldon MC, Ueffing P, Dasgupta ANZ. Estimating progress towards meeting women’s contraceptive needs in 185 countries: A Bayesian hierarchical modelling study. PLoS Med. 2020;17[2]:e1003026. doi: 10.1371/journal.pmed.1003026 32069289 PMC7028249

[4] United Nations Department of Economic and Social Affairs Population Division. Estimates and Projections of Family Planning Indicators 2019. Available from:https://www.un.org/development/desa/pd/content/family-planning-and-2030-agenda-sustainable-. 2020.

[5] Stover J, Ross J. How Increased Contraceptive Use has Reduced Maternal Mortality. Matern Child Health J. 2010;14[5]:687–95. doi: 10.1007/s10995-009-0505-y 19644742

[6] Thomas TN, Gausman J, Lattof SR, Wegner MN, Kearns AD, Langer A. Improved maternal health since the ICPD: 20 years of progress. ICPD Beyond 2014 Expert Meeting on Women’s Health—rights, empowerment and social determinants 30th September - 2nd October, Mexico City. Vol. 90. 2014. doi: 10.1016/j.contraception.2014.06.026 25062996

[7] Ahmed S, Li Q, Liu L, Tsui AO. Maternal Deaths Averted by Contraceptive Use. Population, Family and Reproductive Health Department Johns Hopkins University Bloomberg School of Public Health. Vol. 67. 2012. doi: 10.1016/S0140-6736(12)60478-4 22784531

[8] Chola L, McGee S, Tugendhaft A, Buchmann E, Hofman K. Scaling Up Family Planning to Reduce Maternal and Child Mortality: The Potential Costs and Benefits of Modern Contraceptive Use in South Africa. PLoS One. 2015 Jun;10[6]:e0130077. doi: 10.1371/journal.pone.0130077 26076482 PMC4468244

[9] Hajizadeh M, Nghiem S. Does unwanted pregnancy lead to adverse health and healthcare utilization for mother and child? Evidence from low- and middle-income countries. Int J Public Health. 2020;65:457–68. doi: 10.1007/s00038-020-01358-7 32270238 PMC7275006

[10] Committee on Unintended Pregnancy:Division of Health Promotion and Disease Prevention. Institute of Medicine. (1995). Unintended Pregnancy and the Well-Being of Children and Families. In Brown Sarah S. *and* Eisenberg Leon, *Editors*. GPS World (Vol. 18). 10.1115/1.2005-feb-3.

[11] Yazdkhasti M, Pourreza A, Pirak A, Abdi F. Unintended pregnancy and its adverse social and economic consequences on health system: A narrative review article. Iran J Public Health. 2015;44[1]:12–21. 26060771 PMC4449999

[12] Institute Guttmacher. New Estimates Show Worldwide Decrease in Unintended Pregnancies. 2021. p. 1–9. Available from: https://www.guttmacher.org/news-release/2020/new-estimates-show-worldwide-decrease-unintended-pregnancies.

[13] World Health Organization. Trends in maternal mortality 2000 to 2017: estimates by WHO, UNICEF, UNFPA, World Bank Group and the United Nations Population Division. Geneva: 2019. Licence: CC BY-NC-SA 3.0 IGO. 2019.

[14] Alene M, Yismaw L, Berelie Y, Kassie B, Yeshambel R, Assemie MA. Prevalence and determinants of unintended pregnancy in Ethiopia: A systematic review and meta-analysis of observational studies. PLoS One. 2020;15[4]:e0231012. Available from: doi: 10.1371/journal.pone.0231012 32255774 PMC7138300

[15] Ethiopian MOH. Health Sector Transformation Plan II: 2020/21-2024/25. Vol. 25. 2021.

[16] Ethiopian Public Health Institute (EPHI) [Ethiopia] and ICF. Ethiopia Demographic and Health Survey 2016:Key Indicators. Rockville, Maryland, USA: EPHI and ICF. 2016.

[17] Ethiopian Public Health Institute (EPHI) and ICF. 2019. Mini Demographic and Health Survey 2019: key Indicators. Rockville, Maryland, USA: EPHI and ICF. 2019. 35 p.

[18] Alem AZ, Agegnehu CD. Magnitude and associated factors of unmet need for family planning among rural women in Ethiopia: A multilevel cross-sectional analysis. BMJ Open. 2021;11:e044060. doi: 10.1136/bmjopen-2020-044060 33837100 PMC8043003

[19] Getaneh T, Negesse A, Dessie G, Desta M, Moltot T. Predictors of unmet need for family planning in Ethiopia 2019: a systematic review and meta analysis. Arch Public Heal. 2020;78:102. doi: 10.1186/s13690-020-00483-2 33088503 PMC7566059

[20] Wolde A, Haile D, Anjulo U, Wolde J. Unmet Need for Modern Contraceptive Methods and Associated Factors Among Currently Married Women in Damot Woyde District, SNNPR, Ethiopia, 2019. Open Access J Contracept. 2020;Volume 11:177–85. doi: 10.2147/OAJC.S284129 33262664 PMC7698268

[21] Muche M, Kaso M, Eshete A, Kalayu I. Unmet need for family planning and associated factors among married women in Asebot town, west Hararge zone, Oromia regional state, Ethiopia, Cross- sectional study design, 2018. Glob J Reprod Med. 2019;6[4]:111–7.

[22] G/Meskel AT, Desta HO, Bala ET. Factors Associated with Unmet Need for Family Planning among Married Reproductive Age Women in Toke Kutaye District, Oromia, Ethiopia. Int J Reprod Med. 2021;2021:1–9. doi: 10.1155/2021/5514498 33855065 PMC8019393

[23] Solomon T, Nigatu M, Gebrehiwot TT, Getachew B. Unmet need for family planning and associated factors among currently married reproductive age women in Tiro Afeta District, South West Ethiopia, 2017: Cross-sectional study. BMC Womens Health. 2019;19:170. doi: 10.1186/s12905-019-0872-5 31888594 PMC6937809

[24] Tadele A, Abebaw D, Ali R. Predictors of unmet need for family planning among all women of reproductive age in Ethiopia. Contracept Reprod Med. 2019;4:6. doi: 10.1186/s40834-019-0087-z 31171978 PMC6547515

[25] Worku SA, Mittiku YM, Wubetu AD. Unmet need for family planning in Ethiopia and its association with occupational status of women and discussion to her partner: a systematic review and meta-analysis. Contracept Reprod Med. 2020;5:21. doi: 10.1186/s40834-020-00121-w 33292648 PMC7678115

[26] WHO. Equity–leave no one behind. Monitoring Health For the SDGs. Wold Health Statistics, 2016. 2016. Available from: https://www.who.int/gho/publications/world_health_statistics/2016/EN_WHS2016_Chapter5.pdf.

[27] Worku SA, Ahmed SM, Mulushewa TF. Unmet need for family planning and its associated factor among women of reproductive age in Debre Berhan Town, Amhara, Ethiopia. BMC Res Notes. 2019;12:143. Available from: doi: 10.1186/s13104-019-4180-9 30876437 PMC6419818

[28] Ahinkorah BO, Ameyaw EK, Seidu AA. Socio-economic and demographic predictors of unmet need for contraception among young women in sub-Saharan Africa: evidence from cross-sectional surveys. Reprod Health. 2020;17[1]:1–11. Available from: doi: 10.1186/s12978-019-0847-x 33097088 PMC7585192

[29] Mulenga JN, Bwalya BB, Mulenga MC, Mumba K. Determinants of unmet need for family planning among married women in Zambia. *J Public Health Afr*. 2020;11[1]:1084. Published 2020 Apr 29. doi: 10.4081/jphia.2020.1084 33209230 PMC7649728

[30] Wai MM, Bjertness E, Stigum H, Htay TT, Liabsuetrakul T, Myint ANM, et al. Unmet need for family planning among urban and rural married women in yangon region, myanmar—a cross-sectional study. Int J Environ Res Public Health. 2019;16:3742. doi: 10.3390/ijerph16193742 31590239 PMC6801744

[31] Bishwajit G, Tang S, Yaya S, Feng Z. Unmet need for contraception and its association with unintended pregnancy in Bangladesh. BMC Pregnancy Childbirth. 2017;17:186. doi: 10.1186/s12884-017-1379-4 28606062 PMC5469146

[32] Chafo K, Agide F. Unmet Need for Family Planning and Associated Factors among Currently Married Women in Misha District, Southern Ethiopia: A Cross Sectional Study. J Women’s Heal Care. 2014 Jan 1;03.

[33] Yoonis Ahmed. Assessment of the Magnitude and Determinants of Unmet Need for Family Planning Among Currently Married Women in Reproductive Age in Hargeisa, Somaliland. Int J Soc Sci Humanit Res. 2018;6[4]:1128–43. Available from: https://gollisuniversity.org/wp-content/uploads/2019/02/Assessment-of-the-Magnitude-6832.pdf.

[34] Yadav K, Agarwal M, Shukla M, Singh JV, Singh VK. Erratum: Correction to: Unmet need for family planning services among young married women (15–24 years) living in urban slums of India. BMC Womens Health. 2020;20:187. doi: 10.1186/s12905-020-01010-9 32883262 PMC7469334

[35] Tegegne TK, Chojenta C, Forder PM, Getachew T, Smith R, Loxton D. Spatial variations and associated factors of modern contraceptive use in Ethiopia: a spatial and multilevel analysis. BMJ Open. 2020;10:e037532. doi: 10.1136/bmjopen-2020-037532 33046466 PMC7552846

[36] Gebre MN, Edossa ZK. Modern contraceptive utilization and associated factors among reproductive-age women in Ethiopia: Evidence from 2016 Ethiopia demographic and health survey. BMC Womens Health. 2020;20:61. doi: 10.1186/s12905-020-00923-9 32216823 PMC7098091

[37] Asresie MB, Fekadu GA, Dagnew GW. Contraceptive use among women with no fertility intention in Ethiopia. PLoS One. 2020;15[6]:e0234474. Available from: doi: 10.1371/journal.pone.0234474 32525935 PMC7289351

[38] Rana MJ, Goli S, Mishra R, Gautam A, Datta N, Nanda P, et al. Contraceptive method information and method switching in India. Sustain. 2021;13:9831.

[39] Goli S, Gautam A, Rana MJ, Ram H, Ganguly D, Reja T, et al. Is unintended birth associated with physical intimate partner violence? Evidence from India. J Biosoc Sci. 2020/01/06. 2020;52[6]:907–22. Available from: https://www.cambridge.org/core/article/is-unintended-birth-associated-with-physical-intimate-partner-violence-evidence-from-india/56D579A3F2030C372D74F7531698E98B. doi: 10.1017/S0021932019000865 31902374

[40] Asif MF, Pervaiz Z. Socio-demographic determinants of unmet need for family planning among married women in Pakistan. BMC Public Health. 2019;19:1226. doi: 10.1186/s12889-019-7487-5 31488094 PMC6727575

[41] Dingeta T, Oljira L, Worku A, Berhane Y. Unmet Need for Contraception Among Young Married Women in Eastern Ethiopia. Open Access J Contracept. 2019;Volume 10:89–101. doi: 10.2147/OAJC.S227260 31908548 PMC6925555

[42] Gahungu J, Vahdaninia M, Regmi PR. The unmet needs for modern family planning methods among postpartum women in Sub-Saharan Africa: a systematic review of the literature. Reprod Health. 2021;18:35. Available from: doi: 10.1186/s12978-021-01089-9 33568180 PMC7877117

[43] Gebreegziabher EA, Astawesegn FH, Anjulo AA, Kerie MW. Urban Health Extension Services Utilization in Bishoftu Town, Oromia Regional State, Central Ethiopia. BMC Health Serv Res. 2017;17:195. doi: 10.1186/s12913-017-2129-z 28288620 PMC5348781

[44] Molla S, Tsehay CT, Gebremedhin T. Urban health extension program and health services utilization in northwest Ethiopia: A community-based study. Risk Manag Healthc Policy. 2020;13:2095–102. doi: 10.2147/RMHP.S253847 33116989 PMC7568606

[45] Zebre G, Gizaw AT, Tareke KG, Lemu YK. Implementation, experience, and challenges of urban health extension program in Addis Ababa: a case study from Ethiopia. BMC Public Health. 2021;21:167. doi: 10.1186/s12889-021-10221-0 33468092 PMC7816480

[46] Enden MR, Tolla MT, Norheim OF. Providing universal access to modern contraceptive methods: An extended cost-effectiveness analysis of meeting the demand for modern contraception in Ethiopia. Soc Sci Med. 2021;281:114076. Available from: https://www.sciencedirect.com/science/article/pii/S0277953621004081. doi: 10.1016/j.socscimed.2021.114076 34116422

[47] Black RE, Levin C, Walker N, Chou D, Liu L, Temmerman M. Reproductive, maternal, newborn, and child health: key messages from Disease Control Priorities 3rd Edition. Lancet. 2016 Dec 3;388[10061]:2811–24. Available from: doi: 10.1016/S0140-6736(16)00738-8 27072119

