# Supplementary material for: Unmet need for family planning and associated factors among currently married women of reproductive age in Bishoftu town, Eastern Ethiopia
Source: PLoS One. 2021 Dec 6;16(12):e0260972. doi: 10.1371/journal.pone.0260972 (PMC8648111; doi:10.1371/journal.pone.0260972)
Supplement: S1 File — (ZIP) [file pone.0260972.s001.zip › Supporting Information_Questionnaire/Survey Questionnaire_Afan Oromo.pdf]

GAAFFILLEE AFAAN OROMOON QOPHAAHE

KUTAA I: RAGAA BU'UURA HAWAASUMMAA

001	Lakk. gaaffii	☐ ☐ ☐
002	Iddoo jireenyaa/ Garee/Lakk. manaa	Ganda___________________.<br>Garee/gooxii________________.<br>Lakk. manaa_______________.
003	Guyyaa gaafiif deebii (akka lakk. Ethiopia)	_____/____/ _____ E.C.

Koodi	Gaaaffillee	Deebii	Darbi
**001**	Umuriin kee/keessan meeqaa?	----------- yrs.	
**002**	Amantaan kee/keessan maali?	1=Ortodoksii 2=Waaqeffataa 3=Musliima<br>4=Protestaantii 99=kan biraa, ibsi ____________	
**003**	Sabni kee/keessan malii?	1=Amaara 2=Guraagee 3=Oromoo 4=Tigiree<br>5=Somaalee 99=kan biraa, ibsi __	
**004**	Sadarkaan barumsaa kee hagami?	1=Hin barannee<br>2=Kutaa1-8 ffaa<br>3=Kutaa9-12 ffaa<br>4=Kolleejii fi isaa oli	
**005**	Sadarkaan barumsa abbaa manaa keetii Hagami	1=Hin baranne 2=Kutaa 1-8 ffaa<br>3=Kutaa 9-12 ffaa 4=Kolleejii fi isaa oli	
**006**	Hojiin kee maalii dhaa?	1=Hojii mootummaa 2=Daldaaltuu<br>3=Hojii dhuunfaa 4=Haadha manaa 5=Hojjetuu guyyaa 6=Qotatee bultuu<br>99=Kan biraa ibsi……………	
**007**	Abbaan manaa kee maal hojjeta?	1=Hojii mootummaa 2=Daldalaa<br>3=Hojii dhuunfaa 4=Konkolaachisaa<br>5=Hojjetaa guyyaa 6=Qotee bulaa 99=Kan biraa ibsi……………	
**008**	Galiin ji'aan argattuu qarshii Itiyoopiyaatti haggam ta'a?	--------- Qarshii ji'aan	

KUTAA-II: GAAFILEE WAA'EE FAYYAA WALHORMAATAA ILAALCHISEE

Lakk.	Gaafii	Deebii	Darbii
201	Yeroo dura heerumtu umriin kee/keessan waggaa meeqa?	Waggaa……..	
202	Umrii kee keessatii ulfooftee beektaa?	1= Eeyeen 2=Miti	210
203	Deebiin gaafii lakk. 202 eeyyeen yoo ta'ee yeroo dura ulfooftee sanaa umriin kee meeqa?	Wagga ……..	
204	Ammaa immoo waa'ee daa'iman/ijoollee hangaa ammaa deeseen sii gafaadha. Ijoollee meeqa deesse?	Lakk.………	
206	Ijoollee meeqa qabdaa?	Waliigalatti--------	
207	Hanga Yoonaa daa'imni deessani isin jalaa deebite jirtii/ beektii?	1= Eeye<br>2= Miti/hin jiruu	209
208	Daa'ima dhiraa fi dhalaa meeqatu sii jalaa du'ee?	Lakk.bareessi<br>1=Dhiraa..........<br>2=Dhalaa ....<br>3=Waliigalaatii..............	
209	Yeroo ijoollee homaa hin qabnee(hindeenyee) san gara fulduraatii ijoollee meeqa qabaachuu (dahuu) akkaa barbaadu karoorfatee turte? [waraa ijoollee qaban gaafadhu.]	1=Lakk.bareessii<br>2=Hinbeeku	
210	Waluumaa galaatii ijoollee meeqa qabaachuu barbaada? warra ijoollee hin qabnee gafaadhu]	1=Lakk.bareessii ---------<br>2=Hangaa hoona hinmurteesine	
211	Yeroo amma kana ulfaa?	1=Eeyeen 2=Miti	213
212	Deebiin gaafii lakk.211 yoo eyyee<br>Ta'e ulfi kun ?	1=Ammatii barbaadamaa dha.<br>2=Yeroo murta'eef turun barbaada<br>3=Goonkumaa hin barbaaduun ture	214

213	Ji'oota jahan darbee keessa deesse turtee?	1=Eyyee 2=Miti	223
214	Daa'ima kee dhumaa egaa deesse hangaa ammatii ji'a meeqa ni ta'a?	1=Ji'a__[ji'a 1 bareessii,yoo guyyaa 30 gadii ta'e	
215	Eega deesse booda gara fulduratti daa'ima dabalachuuf ni barbaada? [warra ulfaa gafaadhu]	1=Eeyyen<br>2= Lakkii	219
216	Daa'ima ji'a jahan darbe keessa deesse alatti gara fuulduraatti daa'ima biraa dahuu barbaadaa? [warraa ji'a jahan darbe keessa dahan gaafadhu)	1=Eeyyen<br>2=Lakkii	220
217	Yoo deebiin gaafii lakk.215 eeyyeen yoo ta'e, oso hin dahiin hanga yoomii turuu barbaada?	1=Waggaa lamaa gadii (≤ 2 yrs)<br>2=Waggaa lamaa ol (≤2 yrs)<br>3=Hangaa yoona hinmurteessine	
218	Yoo deebiin gaafii lakk.216 eeyyen yoo ta'e, osoo hin dahiin hanga yoomii turuu barbaada?	1=Waggaa lamaa gadii (≤ 2 yrs)<br>2=Waggaa lamaa ol (>2 yrs)	
219	Ulfa kana eega deesse booda waggaa tokko keessatii karooraa maatii (mala qussaana maatii) fayyadamuu ni barbaaddaa?	1=Eeyeen 2=Miiti 3=Murteesu hin danda'u (nan shakka)	227
220	Ergaa daa'imaa dhuma kana deessee jalqabee waggaa tokko keessatii karoora maattiiti fayyaadamu ni barbaada? [warraa ji'oota jahaan darbee keessa dahaan gafaadhu]	1=Eeyyee<br>2=Miti<br>3=Hanga ammatii hinmurteessine	227
221	Yoo deebiin gaaffii lakk.219 eeyeen ta'ee, mala karoora maatii kana kan fayyadaamtu yeroo murtaa'eef osoo hin ulfa'iin turuuf moo guutuuman guuttutii daahu waan hin barbaaneef?	1=Yeroo murtaayeef turuuf<br>2=Guutumman guuttutii dhala dhaabuu	
222	Yoo deebiin gaaffii lakk.220 eeyeen ta'ee mala karoora matii kana kan fayyadaamtu yeroo murtaa'eef osoo hin ulfa'iin turuuf moo guutuuman guuttutii daahu waan hinbarbaaneef?	1=Yeroo murtaayef turuuf<br>2= Guutumman guuttutii dhala dhaabuuf	

223	Yeroo ammaa kana ulfa yoo hin taane ykn ji’oota jahaan darbee keessaa yoo hin deenye ta’ee garaa fulduraati ijoollee biraa dahuu barbaadaa?	1=Eeyyen<br>2=Lakkii	225
224	Yoo deebiin gaafii lakk.223 eeyyeen yoo ta’ee,daa’ima ittii aanu dahuuf/ ulfaa’uf haga waggaa meeqa turu barbaada?	1=waggaa lamaa gadii<br>2=waggaa lamaa ol<br>3=Hangaa hoona hinmurteessine	
225	Garaa fulduuratii ,jechuun ammara eggale ji’oota 12 keessatii karoora mattitii faayadamuuf karoora qabdaa?	1=Eyeen<br>2=Miti / hin faayadamu /<br>3=Hangaa hoona hinmurteessine	227
227	Maloota karoora maatii keessa kammiiti fayyaadamu barbaada? [Hin dubissinif ,deebii ishiin keenitee hundaatu marrii}	1=Kiiniinii liqmsaamuu(pills)<br>2=Kan ciiqlee harkaa keessa galu (implants/Norplant)<br>3= Kan lilmoodhan waraanaman (injectablesa)<br>4=kan gadaamessaa keessaa ta’u(IUCD)<br>5=koondomii<br>6=kan biiraa (bareessii)_______	

228	Garaa fuldurattii karoora mattitii faayyadaamuu yoo hin barbaadnee ta’ee sababoota tokko tokko natti himuu dandeesuu?<br><br>[Hin duubissiniif, deebii ishiin kennitee hundaatuu marrii]	1=Walqunaamtii saalaa waan hin goneef<br>2=Yeroo hundaa walqunamtii salaa waan hingoneef<br>3=Sabaaba umrii/gadaamessii koo waan opeerrationidhan baheef<br>4=Dhaalu/dahuu waan hindandenyeef<br>5=Guyyaa 45 kana keessa waan daheef<br>6=Waan harmaa hoosisuf<br>7=Daa’imma dabalaata waan barbaaduf<br>8=Mala karoora maatii waan hinbeeknef<br>9=Karoora maatii essaa akka argaadhu waan hinbeeknef<br>10=Waan fayyaadamu hin barbaaneef<br>11=Abbaa warraa/ maatiin koo waan naaf hinhayyaamneef<br>12=Ammaantiin koo hin hayyaamu<br>13=Sababaa fayyaatiin walqabamaan<br>14=Midhaa natii fidaa jedhee waan soodadheef<br>15=Argachuuf rakiisaa ykn idoon ittii argaamu fagoo ykn gonkumaa waan hinargamneef<br>16=Gatiin isaa guddaa waan ta,eef<br>17=Ittii fayyaadamuf natii hin toluu	

KUTAA III: KENNINSA TAJAAJILAAN KAN WALQABATAN

Lakk.	Gaaffii	Deebii	Darbii
229	Bakka karoorri maatii kun itti argamu beektaa?	1=Eyyee<br>2=Lakkii	233
230	Yoo beekte, atii fi namoonni biro eessaa karoora maatii kana argattanii?	1=Hospitaala<br>2=Buufata fayyaa<br>3=Keellaa Fayyaa<br>4=Kiliinika dhuunfaa<br>5=Kan biroo	
231	Karoora maatii kana argachuuf hagam sitti fudhata?	Sa'aatii…………	
232	Karoora maatii kana argachuuf yeroo hagamiif eeggattaa?	1=Daqiiqaa <30<br>2=Daqiiqaa 30--60<br>3=Daqiiqaa >60	
233	Ji'oottan 12n darbanitti Ogeessa fayyaan daaawwatamteettaa?	1=Eyyee<br>2=Lakkii	
234	Filannooan karoora maatii jiran sitti himameeraa?	1=Eyyee<br>2=Lakkii	
235	Yoo eeyyee, jette,filannoo barbaaddu argattee jirtaa?	1=Eyyee<br>2=Lakkii	
236	Beellamni siif laatameeraa?	1=Eyyee<br>2=Lakkii	
