# Supplementary material for: Unmet need for family planning and associated factors among currently married women of reproductive age in Bishoftu town, Eastern Ethiopia
Source: PLoS One. 2021 Dec 6;16(12):e0260972. doi: 10.1371/journal.pone.0260972 (PMC8648111; doi:10.1371/journal.pone.0260972)
Supplement: S1 File — (ZIP) [file pone.0260972.s001.zip › Supporting Information_Questionnaire/Survey Questionnaire_ENGLISH.pdf]

ENGLISH VERSION OF QUESTIONNAIRE

PART I: SOCIODEMOGRAPHICAND ECONOMIC CHARACTERSTICS AMONG STUDY PARTICIPANTS

Code	Questions		
001	Questionnaire number	☐ ☐ ☐	
002	House number /peasant association	Kebele___________________.<br>Peasant Ass.________________.<br>House number_______________.	
003	Date of interview (Ethiopian Calendar)	_____/____/ _____ E.C.	

Code	Questions	Possible Responses	Skip
001	How old are you? Age at interview in completed year	---------- Years	
002	What is your religion?	1. Orthodox 4. Wakefata<br>2. Muslim 3. Protestant<br>99. Others, specify _______	
003	What is your ethnicity?	1. Amhara 3. Gurage<br>2. Oromo 4. Tigre<br>5. Somali 99. Others, specify_____	
004	What is your highest level of education?	1. No education<br>2. 1-8th grade<br>3. 9-12th grade<br>4. College and above grade	
005	What is your husband's highest level of education?	1. No education<br>2. 1-8th grade<br>3. 9-12th grade<br>4. College and above grade	
006	What is your occupation?	1. Government employee<br>2. Self-employee 3. Merchant<br>4 House wife<br>5. Daily laborer 6. Other, specify…	
007	What is your husband's occupational status	1. Government employee<br>2. Self-employee 3. Merchant<br>4. Daily laborer 5. Farmer<br>6. Other, s specifies…	
008	Monthly income in Ethiopian Birr	_________ birr per month	

Part II. REPRODUCTIVE HISTORY OF STUDY PARTICIPANTS

Quest.	Questions	Response	Skip to
201	At what age did you first marry?	_________________years	
202	Have you ever been pregnant?	1=Yes 2=No	210
203	If answer to question no. 202 is yes, how old were you when you first got pregnant?	_________________years	
204	Now I would like to ask you about all the births you have had during your life	Enter number............	
206	Number of living children (Enter total)	__________________	
207	Have you ever given birth to a boy or girl who was born alive but later died?	1=Yes 2=No	209
208	How many sons and daughters have died? (Enter number)	Sons____________<br>Daughter_______<br>Total___________	
209	If you could go back to the time, you don't have children and could choose exactly the number of children to have in your life, how many children could that be?	1=Enter no.______________<br><br>2=I don't know	
210	How many children would you like to have in your life? (for those who don't have children)	1=Enter no _____<br>2=Not yet decided	
211	Are you currently pregnant?	1=Yes 2=No	213
212	If answer to question no.211 is yes, is the pregnancy-----------?	1=Wanted now<br>2=Wanted later<br>3=Not wanted at all	
213	Did you give birth within last six months?	1=Yes<br>2=No	223
214	Time since last birth<br>(for those currently pregnant or give birth in the last 6 month)?	1=______ months<br>2=I don't remember	
215	After the child you are expecting now, would you like to have another child? (for pregnant women)	1=Yes<br>2=No	219
216	After the birth that occurred within the last six months, would you like to have another child (for women that gave birth with in the last six months)	1=Yes<br>2=No	220
217	If the answer to question no.215 is Yes how long would you like to wait before the birth of another child?	1=2years<br>2=> 2 years<br>3=Not yet decided	

218	If the answer to question no.216 is Yes, how long would you like to wait before the birth of another child?	1=2years<br>2=> 2 years<br>3=Not yet decided	
219	Within a year after the birth of the child you are expecting now, will you use modern contraception?<br>(for pregnant women)	1=Yes<br>2=No<br>3=Not sure yet decided	227
220	Do use modern family planning (for women who gave birth with in the last six months)	1=Yes<br>2=No	227
221	If the answer to question.no.219 is yes, would you like to use the method for limiting or for spacing?	1=For spacing<br>2=For limiting	
222	If the answer to question no.219 is yes, for what purpose do you use the method?	1=For spacing<br>2=For limiting	
223	If you are not currently pregnant or didn't give birth in the past six months, would you like to have another child	1=Yes<br>2=No	
224	If the answer to question no.223 is choice 1. How long would you like to wait from now before the birth of another child?	1=2years<br>2=> 2 years<br>3=Not yet decide	
225	Do you use modern contraceptive?<br>(For those neither pregnant nor give birth last 6 months)	1=Yes<br>2=No<br>3=Not yet decided	228
226	For what purpose do you use FP?	1= For spacing<br>2=For Limiting	
227	Which methods do you/would you like to /use? Don't read, Circle all mentioned responses	1=Pills<br>2=Implant /Norplant<br>3=Injectable 4 IUCD<br>4=Condom<br>5=Others, specify	
228 a	If you were not going to use modern contraceptives, would you tell me the main reason? Do not read<br><br>Circle all possible response	1=Not having sex<br>2=Infrequent sex<br>3=Menopausal/hysterectom<br>4=Infertile<br>5=Postpartum amenorrhea<br>6=Breast feeding<br>7=Want more children<br>8=Know no method<br>9=Know no source<br>10=Respondent opposed<br>11=Husband /family opposition<br>12=Religion prohibition	

		13=Health concern<br>14=Fear of side effect<br>15=Lack of access /too far or not available<br>16=Cost too much<br>17=In convenient to use	
228b	If you are not using FP do you want child?	1=Want later<br>2=Not at all	

PART III: SERVICE-RELATED FACTORS

Quest	Question	Response	Skip to
229	Do you know the place where modern? Contraceptive methods could be obtained.	1=Yes<br>2=No	 233
230	If you know where the methods are obtained, where is the main place that you or others are able to get modern Contraceptive?	1=Hospital<br>2=Health center<br>3=Health post<br>4=Private clinic<br>5=Others	
231	How long would it take to reach to the source of contraceptive	………… hours	
232	How long you spend your time to get this contraceptive method.	1=< 30 minutes<br>2=30 to 60 minutes<br>3=> 60 minutes	
233	Have you visited by FP provider in last12 months?	1=Yes<br>2= No	
234	Have you talked about FP options?	2=Yes<br>2=No	
235	If yes, do you get the method you want?	1=Yes<br>2=No	
236	Do you get an appointment?	1=Yes<br>2= No	
