# Supplementary material for: Unmet need for family planning and associated factors among currently married women of reproductive age in Bishoftu town, Eastern Ethiopia
Source: PLoS One. 2021 Dec 6;16(12):e0260972. doi: 10.1371/journal.pone.0260972 (PMC8648111; doi:10.1371/journal.pone.0260972)
Supplement: Attachment — Submitted filename: Response to Editor.docx [file pone.0260972.s003.docx]

31^th^ October, 2021

To Editor

PLOS ONE

Subject: Submitting revised manuscript

It is our great pleasure for the opportunity given to us to submit the revised version of the manuscript. A point-by-point response to the editors’ comments are presented as follows.

1. Can you develop the discussion a little more so that it will be relevant to global readers? Compare your results with other developing countries. For instance, use following references to make a comparative discussion.

**Response:** Thank you very much, we have addressed it.

2. Please ensure that your manuscript meets PLOS ONE's style requirements, including those for file naming.

**Response:** Thank you very much, we have addressed it.

3. Please include additional information regarding the survey or questionnaire used in the study and ensure that you have provided sufficient details that others could replicate the analyses. For instance, if you developed a questionnaire as part of this study and it is not under a copyright more restrictive than CC-BY, please include a copy, in both the original language and English, as Supporting Information.

**Response**: Thank you. We have provided citations indicating list of references used in developing a questionnaire. Additionally, we have supplied the questionnaire as supporting information.

4. You indicated that you had ethical approval for your study. In your Methods section, please ensure you have also stated whether you obtained consent from parents or guardians of the minors included in the study or whether the research ethics committee or IRB specifically waived the need for their consent.

**Response:** Thank you. Informed written consent was taken from parents or guardians for minors as indicated in the revised manuscript (Line # 172-173).

5. In your Data Availability statement, you have not specified where the minimal data set underlying the results described in your manuscript can be found. PLOS defines a study's minimal data set as the underlying data used to reach the conclusions drawn in the manuscript and any additional data required to replicate the reported study findings in their entirety. All PLOS journals require that the minimal data set be made fully available. For more information about our data policy, please see <http://journals.plos.org/plosone/s/data-availability> . Upon re-submitting your revised manuscript, please upload your study’s minimal underlying data set as either Supporting Information files or to a stable, public repository and include the relevant URLs, DOIs, or accession numbers within your revised cover letter. For a list of acceptable repositories, please see http://journals.plos.org/ plosone/s/data-availability# loc-recommended-repositories. Any potentially identifying patient information must be fully anonymized.

**Response:** Thank you. We have supplied the minimal data set as supporting information file.

6. Please review your reference list to ensure that it is complete and correct. If you have cited papers that have been retracted, please include the rationale for doing so in the manuscript text, or remove these references and replace them with relevant current references. Any changes to the reference list should be mentioned in the rebuttal letter that accompanies your revised manuscript. If you need to cite a retracted article, indicate the article’s retracted status in the References list and also include a citation and full reference for the retraction notice.

**Response:** Thank you. We have addressed it.

Kind regards,

Dawit Wolde Daka

Corresponding Author

[dave86520@gmail.com](mailto:dave86520@gmail.com)
